# Supplementary Chromium(III) Propionate Complex Does Not Protect Against Insulin Resistance in High-Fat-Fed Rats

**DOI:** 10.1007/s12011-013-9877-3

**Published:** 2014-01-12

**Authors:** Ewelina Król, Zbigniew Krejpcio, Katarzyna Iwanik

**Affiliations:** 1 Department of Human Nutrition and Hygiene, Poznan University of Life Sciences, Wojska Polskiego 31, 60-624 Poznan, Poland; 2 Department of Clinical Pathology, Poznan University of Medical Sciences, Przybyszewskiego 49, 60-355 Poznan, Poland

**Keywords:** Chromium(III) propionate complex, Insulin resistance, High-fat diet, Rats

## Abstract

Improper eating habits such as high-fat or high-carbohydrate diets are responsible for metabolic changes resulting in impaired glucose tolerance, hyperinsulinemia, insulin resistance, and ultimately diabetes. Although the essentiality of trivalent chromium for humans has been recently questioned by researchers, pharmacological dosages of this element can improve insulin sensitivity in experimental animals and diabetic subjects. The aim of the study was to assess the preventive potential of the supplementary chromium(III) propionate complex (CrProp) in rats fed a high-fat diet. The experiment was conducted on 32 male Wistar rats divided into four groups and fed the following diets: the control (C, AIN-93G), high-fat diets (HF, 40 % energy from fat), and a high-fat diet supplemented with CrProp at dosages of 10 and 50 mg Cr/kg diet (HF + Cr10 and HF + Cr50, respectively). After 8 weeks, high-fat feeding led to an increased body mass, hyperinsulinemia, insulin resistance, a decreased serum urea concentration, accumulation of lipid droplets in hepatocytes, and increased renal Fe and splenic Cu contents. Supplementary CrProp in both dosages did not alleviate these changes but increased renal Cr content and normalized splenic Cu content in high-fat-fed rats. Supplementary CrProp does not prevent the development of insulin resistance in rats fed a high-fat diet.

## Introduction

According to the forecast of the International Diabetes Federation, in the year 2030, the number of diabetics is expected to rise to 552 million worldwide [1]. Studies have shown that insulin resistance, defined as an impaired responsiveness of the body to insulin, is a prediabetic stage associated with obesity, leading to type 2 diabetes [2]; thus, it is believed that early identification and treatment of individuals with prediabetes can delay the progression of full-blown diabetes and related complications [3].

Chromium(III) is a dietary component involved in glucose metabolism, and for over 50 years, it has been considered as an essential element for mammals, including humans. Chromium(III) has a documented role in carbohydrate, lipid, and protein metabolisms [4]. Trivalent chromium has been shown to lower oxidative stress and improve glucose and lipid metabolism; however, the mechanism of its action on the molecular level is not fully understood yet.

Cr(III) supplements have become very popular commercial dietary supplements worldwide, advertised for reducing body mass and improving blood glucose control, especially for diabetic subjects. However, most of these claims were not supported in well-controlled studies. Recently, the “essentiality” of chromium has been questioned due to its ubiquitous nature, very low dietary requirement [5], and a lack of a reliable marker [6].

The major doubt came from the results of a study performed in Vincent’s laboratory [5]. The authors fed male Zucker lean rats the AIN-93G diet with no added chromium in the mineral mix component of the diet (low-Cr diet—16 μg/kg), the standard AIN-93G diet (1,135 μg/kg), the standard AIN-93G diet supplemented with 200 μg Cr/kg (1,331 μg/kg), or the standard AIN-93G diet supplemented with 1,000 μg Cr/kg (2,080 μg/kg) for 6 months. It turned out that the chromium content of the diet had no effect on body mass or food intake, and serum glucose levels in glucose tolerance or insulin tolerance tests. The studies revealed that a diet with as little chromium as reasonably possible had no effect on body composition, glucose metabolism, or insulin sensitivity compared with a chromium-“sufficient” diet. Based on these observations, the authors concluded that chromium can no longer be considered an essential element.

The history of biochemical and nutritional studies of Cr has been broadly reviewed by Vincent and Love [7] and will not be repeated in detail in this article.

If Cr is not an essential element for mammals, but at certain dosages modulates glucose and lipids homeostasis, its actions, still not fully understood, could be called “pharmacological.”

Among the various chemical forms of Cr(III) including those used in dietary supplements, the Cr(III) propionate complex (CrProp) [Cr_3_O(O_2_CCH_2_CH_3_)_6_(H_2_O)_3_]^+^, also called Cr3, is of particular interest. Most results concerning the properties and biological activity of this compound come from Vincent’s laboratory [8–10].

In our previous studies [11–13], we showed using various animal models that CrProp, given at dosages of 1 and 5 mg/kg body mass (b.m.)/day, has a significant insulin-sensitizing antidiabetic potential. The therapeutic properties of CrProp in relation to its antidiabetic, insulin-sensitizing potential were discussed in previous publications [9, 11, 12].

The objective of this study was to examine the preventive potential of supplementary CrProp, given at dosages of 10 and 50 mg Cr/kg diet, against insulin resistance induced naturally by feeding rats a high-fat diet.

## Materials and Methods

### Animals and Diets

The experiment was carried out according to the Animal Welfare Standards. All the procedures used in this study were accepted by the Animal Bioethics Committee of Poznan, Poland (approval no. 59/2005).

Eight-week-old male Wistar rats (*n* = 32) with body weight ranging from 188 to 248 g were purchased at the Licensed Laboratory Animal Breeding Center (Poznan, Poland). During both the adaptation and the experimental period, animals were housed under controlled temperature (21 ± 2 °C), humidity (55–60 %), and with a 12 h/12 h day/night cycle. After 5 days of the adaptation period, animals were divided into four groups of eight rats each and kept individually in metal-free individual cages. Animals were fed ad libitum standard and high-fat diets composed according to the AIN-93G recommendations [14] of casein (20 %), sunflower oil (7 %), wheat starch (53.2 %), sucrose (10 %), potato starch (5 %), l-cysteine (0.3 %), vitamin mix AIN-93M (1 %), and mineral mix AIN-93M (3.5 %). The high-fat diets (HF) were obtained from the basal AIN-93 diet, by replacement of wheat starch with fat (up to 25 %, approximately 40 % energy from fat). Supplementary Cr was given in the form of CrProp at dosages of 10 and 50 mg Cr/kg diet, so the diets had variable levels of Cr, such as C (control, 2 mg Cr/kg), HF (high fat, 2 mg Cr/kg), HF + Cr10 (high fat, 10 mg Cr/kg), and HF + Cr50 (high fat, 50 mg Cr/kg). Diets were prepared once a week and stored in a refrigerator. Food intake was monitored daily and body mass gain weekly. The chemical composition of experimental diets is presented in Table 1.

**Table 1:** Chemical composition of diets used in experiment (mean ± SD)

Ingredient	C	Diets		
		HF	HF + Cr10	HF + Cr50
Energy (MJ, 100 g)	1.83 ± 0.01	2.27 ± 0.02	2.28 ± 0.03	2.29 ± 0.02
Protein (%)	18.7 ± 1.23	17.5 ± 0.14	17.7 ± 036	17.9 ± 0.13
Fat (%)	7.14 ± 0.31	25.4 ± 1.07	26.4 ± 0.21	26.4 ± 0.08
Carbohydrates (%)	61.7	47.9	46.9	46.2
Dry mass (%)	91.8 ± 0.19	93.7 ± 0.31	94.0 ± 0.32	93.5 ± 0.18
Ash (%)	4.06 ± 0.71	2.89 ± 0.23	2.99 ± 0.11	2.90 ± 0.12
Cr (μg/g)	2.01 ± 0.51	2.10 ± 0.28	10.1 ± 0.79	50.4 ± 4.71

*C* control (AIN-93G), *HF* high-fat, *HF + Cr10* high-fat with Cr (10 mg/kg diet), *HF + Cr50* high-fat with Cr (50 mg/kg diet)

### Test Chemicals

The source of supplemental Cr(III) was CrProp (chemical formula [Cr_3_O(O_2_CCH_2_CH_3_)_6_(H_2_O)_3_]^+^(NO_3_), called Cr3), added to the mineral mix to obtain 1, 10, and 50 mg Cr/kg diet. The compound was synthesized at the laboratory of the Department of Product Ecology, Poznan University of Economics, according to the method described previously by Earnshaw et al. [15]. The content of Cr in mineral mix and diets was assured by the atomic absorption spectroscopy (AAS) method (an AAS-3 spectrometer with background correction (BC), Zeiss, Germany). The authenticity was established using the physicochemical characteristics of CrProp as described previously [16]. The addition of CrProp to diets has no impact on feed palatability.

### Data Collection

At the end of the experiment, after 16-h fasting, rats were anesthetized with an intraperitoneal thiopental injection (35 mg/kg b.m.) and dissected to collect blood from the aorta and remove inner organs (liver, kidneys, heart, spleen, pancreas, testes) for appropriate biochemical tests. Organs were washed in saline, weighed, and stored at −20 °C until analyzed.

### Blood Biochemistry

Blood serum indices were determined by the following methods: glucose concentration by the hexokinase method [17] and total, LDL, and HDL cholesterol contents and triacylglycerol (TAG) concentrations by the colorimetric methods [18–20] using Olympus AU 560 equipment. Plasma insulin concentration was measured by the RIA method using a kit specific for rats (Linco Research, St. Charles, MO, USA).

Activity of alanine transaminase (ALT) and aspartate transaminase (AST) enzymes was measured by the kinetic methods [21], while urea concentration by the kinetic method using urease and glutamine dehydrogenase, and creatinine concentration by Jaffe’s kinetic method with picric acid [22]. The total protein concentration was determined by the colorimetric method using Cu^+2^ ions [23].

The efficacy of glucose utilization and insulin resistance was characterized by the homeostasis model assessment (HOMA) indices [24].

$$ \mathrm{HOMA}\hbox{-} \mathrm{IR}=\left(\mathrm{Fasting}\ \mathrm{glucose}\left[\mathrm{mmol}/{\mathrm{dm}}^3\right]\times \mathrm{Fasting}\ \mathrm{insulin}\ \left[\mathrm{mIU}/{\mathrm{dm}}^3\right]\right)/22.5 $$

### Histology of the Liver and the Kidney

A 3- to 5-mm section of the representative tissue (liver and kidney) was collected at the time of sacrifice and preserved in 10 % buffered formalin. Sections were processed by standard histologic methods and embedded in paraffin. Slices were prepared and stained with hematoxylin and eosin [25].

### Microelement Determinations

Organs were digested in 65 % (*w*/*w*) spectra pure HNO_3_ (Merck) in the Microwave Digestion System (MARS 5, CEM). Then, the concentrations of Fe, Zn, and Cu in the mineral solutions were measured by the F-AAS method (AAS-3 spectrometer, Zeiss, with BC, Germany), while Cr content was determined by the graphite furnace AAS method (AA EA 5 spectrometer, with BC, Jenoptic, Germany).

The accuracy of quantitative determinations of Fe, Zn, Cu, and Cr was assured by simultaneous analyses of the certified reference material (Pig Kidney BCR® No. 186, Brussels, fortified with Cr standard).

### Statistical Analysis

All results are presented as arithmetic means ± standard deviation, and significance of differences in means was calculated using the one-way ANOVA and Tukey’s test. Means were considered statistically different at *p* < 0.05. All calculations were made using the STATISTICA (version 7.0) program (StatSoft, Inc., Tulsa).

## Results

### General Growth Indices

Feeding rats with high-fat diets resulted in the slightly increasing body weight gain and the significantly increasing body mass/body length ratio (by 10 %), but it did not affect relative internal organ masses. Generally, supplementary CrProp did not affect overall growth indices in rats fed a high-fat diet; however, in CrProp-supplemented rats, relative liver mass tended to decrease (Table 2).

**Table 2:** Effect of CrProp supplementation on overall growth indices in rats (mean ± SD)

Index	Experimental group			
	C	HF	HF + Cr10	HF + Cr50
Food intake (g/day)	20.86 ± 1.59	19.19 ± 0.49	19.09 ± 0.49	18.87 ± 1.74
Body mass gain (g)/56 days	210 ± 21	245 ± 29	252 ± 18	250 ± 28
Body mass/body length ratio (g/cm)	17.3 ± 1.23 a	19.0 ± 0.91 b	18.6 ± 0.81 b	18.3 ± 1.32 b
Liver (% b.m.)	2.86 ± 0.16	2.99 ± 0.29	2.88 ± 0.18	2.80 ± 0.09
Kidneys (% b.m.)	0.61 ± 0.07	0.60 ± 0.07	0.55 ± 0.03	0.57 ± 0.03
Spleen (% b.m.)	0.16 ± 0.02	0.15 ± 0.02	0.17 ± 0.02	0.17 ± 0.03
Heart (% b.m.)	0.29 ± 0.02	0.29 ± 0.04	0.32 ± 0.01	0.30 ± 0.03
Testes (% b.m.)	0.85 ± 0.10	0.90 ± 0.14	0.89 ± 0.11	0.90 ± 0.08
Pancreas (% b.m.)	0.33 ± 0.05	0.32 ± 0.03	0.33 ± 0.05	0.37 ± 0.05

Means on the same line without a common lowercase letter differ significantly (*p* < 0.05)

*C* control (AIN-93G), *HF* high-fat fed, *HF + Cr10* high-fat with Cr (10 mg/kg diet), *HF + Cr50* high-fat with Cr (50 mg/kg diet)

### Insulin Resistance and Lipid Indices

Table 3 presents the effects of high-fat diet and supplementary CrProp on biochemical indices in rats. Feeding rats a high-fat diet (40 % energy from fat) did not affect serum lipid indices or glucose levels, but it induced insulin resistance as evidenced by the twofold increased serum insulin level and homeostasis model assessment-estimated insulin resistance (HOMA-IR) index. Supplementary CrProp (in both dosages) did not influence glucose metabolic indices (glucose, insulin level, and HOMA-IR index) in rats fed high-fat diets. Serum lipid profile was also unchanged, with exception to serum TAG that tended to decrease in CrProp-supplemented groups.

**Table 3:** Effect of high-fat diet and supplemental CrProp on blood serum lipids indices in rats (mean ± SD)

Parameter	Experimental group			
	C	HF	HF + Cr10	HF + Cr50
Glucose (mmol/dm^3^)	8.01 ± 0.76	8.35 ± 0.67	8.27 ± 0.75	8.09 ± 0.71
Insulin (mU/dm^3^)	29.7 ± 13.2 a	59.3 ± 20.4 b	58.9 ± 16.6 b	55.2 ± 20.8 b
HOMA-IR	10.6 ± 4.90 a	22.3 ± 8.71 b	20.6 ± 6.62 b	20.3 ± 8.44 b
Total cholesterol (mmol/dm^3^)	2.49 ± 0.33	2.36 ± 0.24	2.39 ± 0.28	2.23 ± 0.25
HDL cholesterol (mmol/dm^3^)	1.69 ± 0.23	1.64 ± 0.14	1.59 ± 0.17	1.52 ± 0.25
LDL cholesterol (mmol/dm^3^)	0.43 ± 0.09	0.37 ± 0.04	0.38 ± 0.05	0.38 ± 0.08
Triacylglycerols (mmol/dm^3^)	1.63 ± 0.11	1.61 ± 0.68	1.48 ± 0.35	1.45 ± 0.68

Table notes and abbreviations the same as in Table 2

### Toxicity Indices and Histology of the Liver and the Kidney

Feeding neither a high-fat diet nor supplementary CrProp (in both dosages) significantly affected indices of liver damage (serum ALT and AST activity) or serum creatinine levels, whereas the high-fat diet significantly decreased serum urea concentration by 16 %. Supplementary CrProp did not affect these indices in rats fed high-fat diets (Table 4).

**Table 4:** Effect of high-fat diet and supplemental CrProp on blood toxicity markers in rats (mean ± SD)

Blood index	Experimental group			
	C	HF	HF + Cr10	HF + Cr50
ALT (U/dm^3^)	19.0 ± 2.32	23.6 ± 6.32	23.3 ± 3.41	20.5 ± 1.91
AST (U/dm^3^)	97.3 ± 12.7	100.2 ± 15.9	107.0 ± 9.9	98.3 ± 10.8
Total protein (10^−2^ kg/dm^3^)	6.44 ± 0.37	6.38 ± 0.29	6.17 ± 0.15	6.15 ± 0.20
Creatinine (μmol/dm^3^)	36.2 ± 3.52	36.2 ± 3.51	35.4 ± 0	36.2 ± 3.54
Urea (mmol/dm^3^)	5.63 ± 0.77	4.75 ± 0.93	4.25 ± 0.37	4.39 ± 0.52

Abbreviations the same as in Table 2

**Fig. 1:**
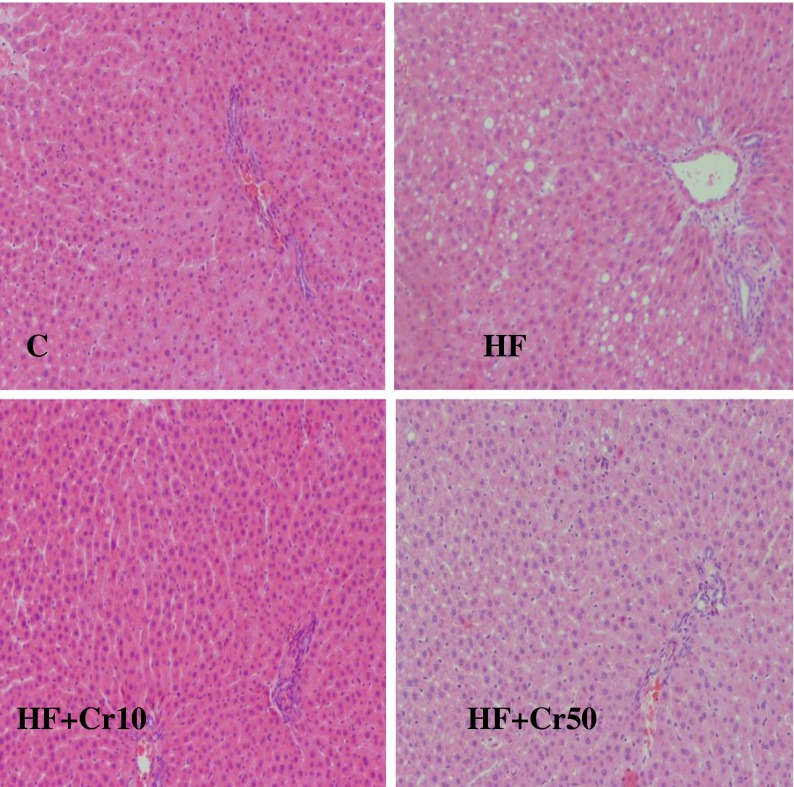
Histopathological images of rats’ livers (zoom ×750): C—control group, HF—group fed high-fat diet, HF + Cr10—group fed high-fat diet supplemented with 10 mg Cr/kg diet, and HF + Cr50—group fed high-fat diet supplemented with 50 mg Cr/kg diet

**Fig. 2:**
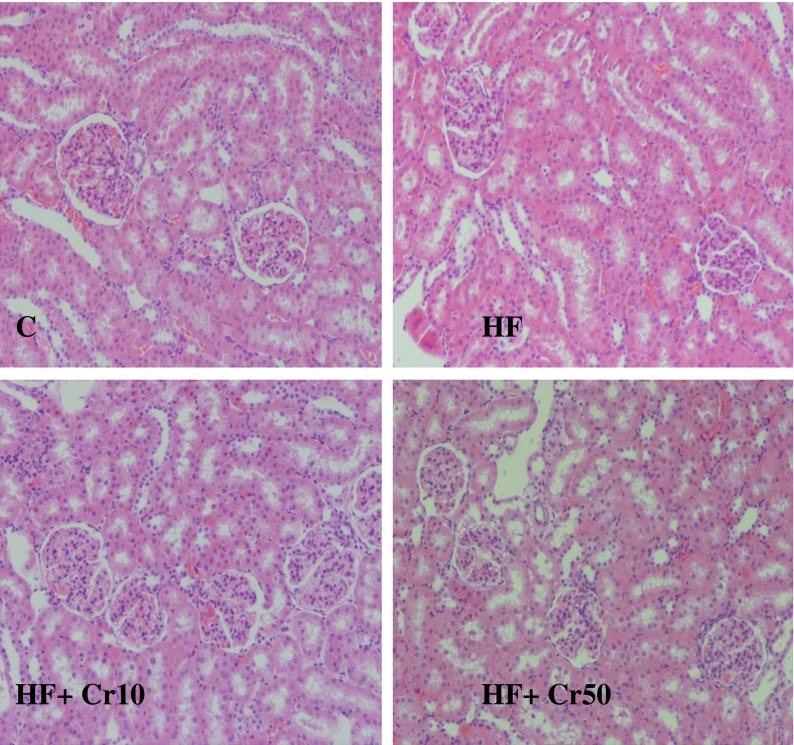
Histopathological images of rats’ kidney (zoom ×750): C—control group, HF—group fed high-fat diet, HF + Cr10—group fed high-fat diet supplemented with 10 mg Cr/kg diet, and HF + Cr50—group fed high-fat diet supplemented with 50 mg Cr/kg diet

The morphological structures of the liver and the kidney are presented in Figs. 1 and 2. Feeding high-fat diets (40 % energy from fat) led to the accumulation of lipid droplets in hepatocytes but without symptoms of inflammation, necrosis, or sclerotization in the glomerulus.

Neither the interstitium nor glomerulus of the kidney was affected by feeding rats with a high-fat diet. Supplementary CrProp slightly diminished liver lipid accumulation and did not influence kidney morphological structures in rats fed high-fat diets.

### Tissue Mineral Content

Feeding rats with a high-fat diet increased renal Fe content and splenic Cu content by 29 and 16 %, respectively, but it did not affect Zn and Cr contents in tissues in comparison with the control (Table 5). Supplementary CrProp normalized or even slightly decreased splenic Cu content, but it did not affect Fe, Zn, or Cu levels in the other internal organs.

**Table 5:** Effect of high-fat diet and supplemental CrProp on the tissular iron, zinc, copper, and chromium levels in rats (mean ± SD)

Index	Experimental group			
	C	HF	HF + Cr10	HF + Cr50
Fe (μg/g dry matter (d.m.))				
Liver	359 ± 43.1	372 ± 53.3	339 ± 47.4	393 ± 61.0
Kidney	280 ± 40.1 a	360 ± 49.01 b	379 ± 35.2 b	363 ± 39.1 b
Spleen	2,560 ± 517	2,551 ± 538	2,597 ± 198	2,765 ± 594
Zn (μg/g d.m.)				
Liver	90.1 ± 4.25	96.6 ± 8.12	90.2 ± 9.23	96.9 ± 6.03
Kidney	86.7 ± 3.55	92.2 ± 5.91	93.8 ± 7.65	96.1 ± 6.47
Spleen	41.37 ± 4.26	41.45 ± 7.24	46.04 ± 3.02	48.05 ± 4.08
Cu (μg/g d.m.)				
Liver	15.3 ± 2.91	15.8 ± 0.63	13.7 ± 1.45	15.9 ± 0.81
Kidney	20.1 ± 2.13	24.6 ± 4.32	24.6 ± 4.78	24.4 ± 4.23
Spleen	8.35 ± 2.79 a	9.67 ± 3.25 b	6.01 ± 0.95 a	6.06 ± 1.00 a
Cr (μg/g d.m.)				
Liver	0.62 ± 0.18 a	0.58 ± 0.21 a	0.92 ± 0.53 ab	1.19 ± 0.64 b
Kidney	0.46 ± 0.17 a	0.52 ± 0.23 a	1.54 ± 0.7 b	2.83 ± 0.58 c

Table notes and abbreviations the same as in Table 2

As it could have been expected, supplementary CrProp (10 and 50 mg Cr/kg diet), given in 5- and 25-fold higher dosages than the control, increased tissular Cr contents; in the liver, it increased by 48 and 105 %, respectively, while in the kidneys, it increased by 196 and 444 %, respectively. The degree of the increase of Cr content in internal organs was moderate, and kidneys were the main target tissue.

## Discussion

A number of studies both on diabetic animals and diabetic patients reported that Cr(III) supplementation may be beneficial, as evidenced by a decreased blood glucose, glycosylated hemoglobin, and cholesterol values or decreased insulin requirements after chromium supplementation [11, 13, 26–29]. On the other hand, there are also clinical trials showing that supplementary Cr(III) did not improve significantly blood biochemistry indices [30–33]. The exact mechanism of Cr action has not been fully elucidated to date.

It is believed that the efficiency of supplementary Cr in the regulation of disturbed glucose and lipid metabolism depends on various factors, such as the chemical form and dose of Cr (and its bioavailability), the degree of insulin resistance or hyperglycemia, and individual sensitivity and genetics, which are not fully defined factors.

The chromium(III) propionate complex (CrProp) was found to be a promising insulin-activating agent, due to its high stability and solubility in the physiological milieu and high absorption rate reaching up to 40–60 % [8]. In a previous study by a team of Vincent and coworkers [10], this compound given to rats showed insulin-sensitizing properties. Lower dosages of CrProp (20 μg/kg b.m./day) after a 12-week administration resulted in lowering of blood plasma LDL, total, and HDL cholesterol levels and triglyceride levels in rats. In another study performed by that team [34], Zucker obese rats (a model of the early stages of type 2 diabetes), CrProp lowered fasting plasma total, HDL, and LDL cholesterol, triglyceride concentrations, as well as insulin levels, and it lowered 2-h plasma insulin levels after 24 weeks of administration. On the other hand, this compound had little effect on rats with streptozotocin (STZ)-induced diabetes (a type 1 diabetes model). These results were further confirmed by Clodfelder et al. [9], who applied higher dosages of CrProp (up to 1,000 μg Cr/kg b.m./day) in healthy and type 2 diabetic rats. It is worth noting that this study was conducted on rats, where the metabolic changes associated with diabetes were induced pharmacologically or by genetic engineering. In those models, hyperglycemia and insulin resistance were more pronounced; thus, the effects of CrProp were also more pronounced.

In this study, we tested the preventive potential of supplementary Cr, given in the form of CrProp, against insulin resistance in Wistar rats. There are different ways of inducing insulin resistance and diabetes in experimental animals, including genetic modification (e.g., obese animals), pharmacologic intervention (e.g., STZ and alloxan injection), dietary manipulation (e.g., high-fat and high-fructose diets), and their combinations (e.g., high-fat feeding and STZ injection). Some studies [35] showed that a high-fat diet (~65 % calories from fat) is more effective than a high-fructose diet (~65 % calories from fructose) in inducing insulin resistance in Wistar rats.

The impact of different fat types on glucose and lipid-related parameters was excessively studied by Buetner et al. [36]. Those authors confirmed that the rats administered a 12-week lard-based high-fat diet (approximately 42 % energy from fat) gave symptoms resembling the human metabolic syndrome, i.e., obesity, adiposity, an increased insulin resistance index (HOMA-IR), or decreased serum adiponectin levels, but not inflammation. Another way of inducing insulin resistance is feeding animals a high-fructose diet (up to 60 %), which leads to increased adiposity and free fatty acid contents in adipose tissues [37], hypertension, oxidative stress, increased hydrogen peroxide generation, and inflammation [38–40]. Generally, both animal models of insulin resistance appear to be suitable for testing insulin-sensitizing agents.

In the present study, we used a high-fat diet to induce insulin resistance in rats. There are significant differences in lipid metabolism in rats and humans. These rodents show higher tolerance to excessive fat consumption in comparison to human subjects; however, this method seems more natural in mimicking the process that occurs in humans than the pharmacologic intervention.

Forbes et al. [41] investigated the effect of different diets, which could be classified as Western diets, on insulin resistance. It was found that excessive consumption of saturated animal origin fat increased body mass and adiposity, decreased insulin sensitivity, and impaired skeletal muscle insulin signaling and insulin hypersecretion. The observed effect depends not only on the total amount of fat, but first of all, on the fatty acid composition of dietary components [36]. High-fat diet induces liver steatosis and disturbs iron metabolism in rats [42].

In the last few years, several publications appeared regarding the influence of supplementation with Cr(III) compounds on high-fat-fed animals. For example, Sahin et al. [43] compared the effects of two Cr(III) compounds, i.e., Cr glycinate (CrGly) and Cr acetate (CrAc), given at dosages of 4 and 8 μg/kg b.m./day on Wistar rats fed high-fat diets. It was found that animals receiving CrGly had greater serum insulin concentrations (by 3.0 %), while those treated with CrAc had lower serum glucose concentrations (by 1.7 %) as well as the glucose/insulin ratio (by 4.49 %). Similar results were observed for Cr(III) histidinate (CrHis) [44].

Kandadi et al. [45] reported that supplementary chromium (d-phenylalanine)_3_ (45 μg Cr/kg b.m./day) had no effect on food intake and body and organ masses, but it normalized serum glucose, insulin, and triglyceride levels in C57BL/6 mice fed high-fat diets.

Chen et al. [46] studied the effect of supplementary Cr-containing milk concentrate capsule (80 μg/kg b.m./day) and high-fat diets (67 % of calories provided by fat) on KK/HIJ mice. It was found that supplementary Cr reduced the hepatic triglyceride accumulation, elevated hepatic lipid catabolic enzymes, improved liver histopathological changes, and suppressed inflammation as well as oxidative stress. Similar improvements in liver histology were also observed by Sahin et al. [47] in high-fat diet fed/STZ-injected Sprague–Dawley rats supplemented with Cr(III) picolinate (80 μg/kg b.m.). In this study, CrProp supplementation tended to decrease relative liver mass and serum triglyceride concentration and slightly improved liver histopathological changes caused by high-fat feeding. These results suggest that this compound slightly inhibits the liver steatosis; however, the liver triglyceride content was not determined, and therefore, it needs to be confirmed in a further study.

In our previous studies [11, 12], insulin resistance was induced by feeding rats for 8 weeks a high-fructose diet (60 % fructose w/w) and a high-fat diet (40 % calories) and by STZ injection (35 mg/kg b.m.). However, these models gave different degrees of metabolic changes in terms of insulin resistance and related effects in rats.

In particular, the first treatment induced insulin resistance without hyperglycemia (HOMA-IR index increased by 230 %, with only a slight increase of serum glucose by 16 %), while the latter model resulted in insulin resistance (HOMA-IR index increased by 130 %) with hyperglycemia (serum glucose increased by 170 %). The therapeutic potential of CrProp, given at the dosages of 1 and 5 mg Cr/kg b.m./day, was then tested on those models. It was found that in high-fructose-fed rats, supplementary CrProp in both dosages was able to significantly decrease serum insulin levels and insulin resistance indexes HOMA-IR and HOMA-B, while it increased the QUICKI insulin sensitivity index. Also, in high-fat/STZ rats, CrProp, especially given in a higher dosage, was more effective in improving those indices. The efficiency of supplementary Cr depended, among other factors, on the degree of metabolic disturbances occurring in the organism, in the following mode: the higher the degree of insulin resistance or hyperglycemia, the stronger the effect of Cr, which suggests its pharmacological action.

This statement falls in agreement with the observations that supplementary Cr, no matter what chemical form or dosage is applied, has no effect on a healthy organism, both in animals and humans. Recently, Herring et al. [48] studied the same Cr compound (Cr3 or CrProp) in healthy rats fed cafeteria-style diets (high-fat and high-carbohydrate diets) in a long exposure experiment. After a 15-month feeding of rats using those diets, CrProp at a dosage of 1 mg/kg b.m./day had no effect on body mass, organ masses (liver, kidneys, lungs, and heart), or visceral fat mass. In the present study, the calculated average daily Cr intake in experimental CrProp-supplemented groups was 0.58 and 2.85 mg Cr/kg b.m./day, respectively (based on the daily food intake, analyzed Cr content in the diet, and weekly body gain).

Cr in its trivalent form can interact in vivo with other essential elements, especially with Fe. The Fe–Cr interaction may occur, since these elements have the same transport protein, transferrin. The relationship between an increased dietary Cr(III) intake and Fe stores was confirmed in several studies. For example, in our previous study, 8-week CrProp supplementation (1 mg Cr/kg b.m./day) decreased Fe kidney concentration [11]; in contrast, a 4-week administration of CrProp in the same dosages did not influence the examined parameters of iron status in rats fed a high-fructose diet [49]. In obese type 2 diabetic rats, supplementation of 1,000 μg Cr/kg b.m./day in the form of CrProp reduced kidney Fe level [12]. Consistent results were observed for the same dosages used in another experiment, where CrProp normalized an increased liver Fe content in rats fed high-fat diets/STZ-injected rats [12]. On the other hand, another Cr complex, CrHis, did not change tissular Fe concentration in a similar animal model of type 2 diabetes after 10 weeks of supplementation [50].

The effects of different Cr compounds on mineral metabolism have been extensively investigated by Staniek et al. [51]. In that comparative study, supplementary Cr (1 mg/kg b.m./day), given in the form of CrProp or CrCl_3_ for 12 weeks, significantly increased Cr accumulation in kidneys of lean and obese but not Zucker diabetic rats. Generally, there were no changes in tissular contents of Ca, Mg, Fe, and Zn, except for Cu, in rats.

Similarly, supplementary CrProp affected Cu levels in rats fed a high-fructose diet for 4 or 8 weeks. In particular, this compound normalized elevated liver and splenic Cu contents [11, 49], while it decreased hepatic and renal Cu levels in diabetic rats [12].

In conclusion, this study showed that supplementary CrProp, given in the dosages of 10 and 50 mg Cr/kg diet (approximately 0.6 and 3 mg Cr/kg b.m./day, respectively), is not able to prevent insulin resistance induced by feeding rats with a high-fat diet.
